# Swimming, Feeding, and Inversion of Multicellular Choanoflagellate Sheets

**DOI:** 10.1103/PhysRevLett.131.168401

**Published:** 2023-10-20

**Authors:** Lloyd Fung, Adam Konkol, Takuji Ishikawa, Ben T. Larson, Thibaut Brunet, Raymond E. Goldstein

**Affiliations:** 1 Department of Applied Mathematics and Theoretical Physics, Centre for Mathematical Sciences, University of Cambridge, Wilberforce Road, Cambridge CB3 0WA, United Kingdom; 2 Department of Biomedical Engineering, Tohoku University, 6-6-01 Aoba, Aramaki, Aoba-ku, Sendai 980-8579, Japan; 3 Department of Biochemistry and Biophysics, University of California, San Francisco, 600 16th Street, San Francisco, California 94143-2200, USA; 4 Institut Pasteur, Université Paris-Cité, CNRS UMR3691, Evolutionary Cell Biology and Evolution of Morphogenesis Unit, 25-28 rue du Docteur Roux, 75015 Paris, France

## Abstract

The recent discovery of the striking sheetlike multicellular choanoflagellate species *Choanoeca flexa* that dynamically interconverts between two hemispherical forms of opposite orientation raises fundamental questions in cell and evolutionary biology, as choanoflagellates are the closest living relatives of animals. It similarly motivates questions in fluid and solid mechanics concerning the differential swimming speeds in the two states and the mechanism of curvature inversion triggered by changes in the geometry of microvilli emanating from each cell. Here we develop fluid dynamical and mechanical models to address these observations and show that they capture the main features of the swimming, feeding, and inversion of *C. flexa* colonies, which can be viewed as active, shape-shifting polymerized membranes.

Some of the most fascinating processes in the developmental biology of complex multicellular organisms involve radical changes in geometry or topology. From the folding of tissues during gastrulation [1] to the formation of hollow spaces in plants [2], these processes involve cell shape changes, cell division, migration and apoptosis, and formation of an extracellular matrix (ECM). Multiple strands of research have shown evolutionary precedents for these processes in some of the simplest multicellular organisms such as green algae [3,4] and choanoflagellates [5], the latter being the closest living relatives of animals. Named for their funnel-shaped collar of microvilli that facilitates filter feeding from the flows driven by their beating flagellum, choanoflagellates serve as model organisms for the evolution of multicellularity.

While well-known multicellular choanoflagellates exist as linear chains or “rosettes” [6] held together by an ECM [7], the new species *Choanoeca flexa* was recently discovered [8] with an unusual sheet-like geometry (Fig. 1) in which hundreds of cells adhere to each other by their microvilli tips, without an ECM [9]. The sheets exist in two forms with opposite curvature, one with flagella pointing towards the center of curvature (“*flag-in*”) with a relatively large spacing between cells, and another with the opposite arrangement (“*flag-out*”) with more tightly packed cells. Transformations between the two can be triggered by darkness, and occur in ~10 s. Experiments [8] show that the *flag-in* state has very limited motility, but greatly enhanced filter feeding. In laboratory conditions, the darkness-induced transition to the more motile *flag-out* form allows a type of photokinesis [8].

As a first step toward understanding principles that govern such a novel organism as *C. flexa*, we analyze two models for these shape-shifting structures. The fluid mechanics are studied by representing the cell raft as a collection of spheres distributed on a hemispherical surface, with nearby point forces representing the action of flagella. Such a model has been used to describe the motility of small sheetlike multicellular assembles such as the alga *Gonium* [13]. The motility and filtering flow through these rafts as a function of cell spacing and curvature explain the observed properties of *C. flexa*. Abstracting the complex elastic interactions between cells to the simplest connectivity, we show how linear elasticity at the microscale controls the competition between two sheet curvatures of opposite sign. These results show that *C. flexa* is a remarkable example of an *active* “polymerized membrane” [14], whose elasticity and dynamics can be studied on accessible length scales and timescales, including the role of topological defects in the large-scale shapes, and fundamental questions in the fluid dynamics of porous structures.

## Fluid mechanics of feeding and swimming

**Fig. 1:**
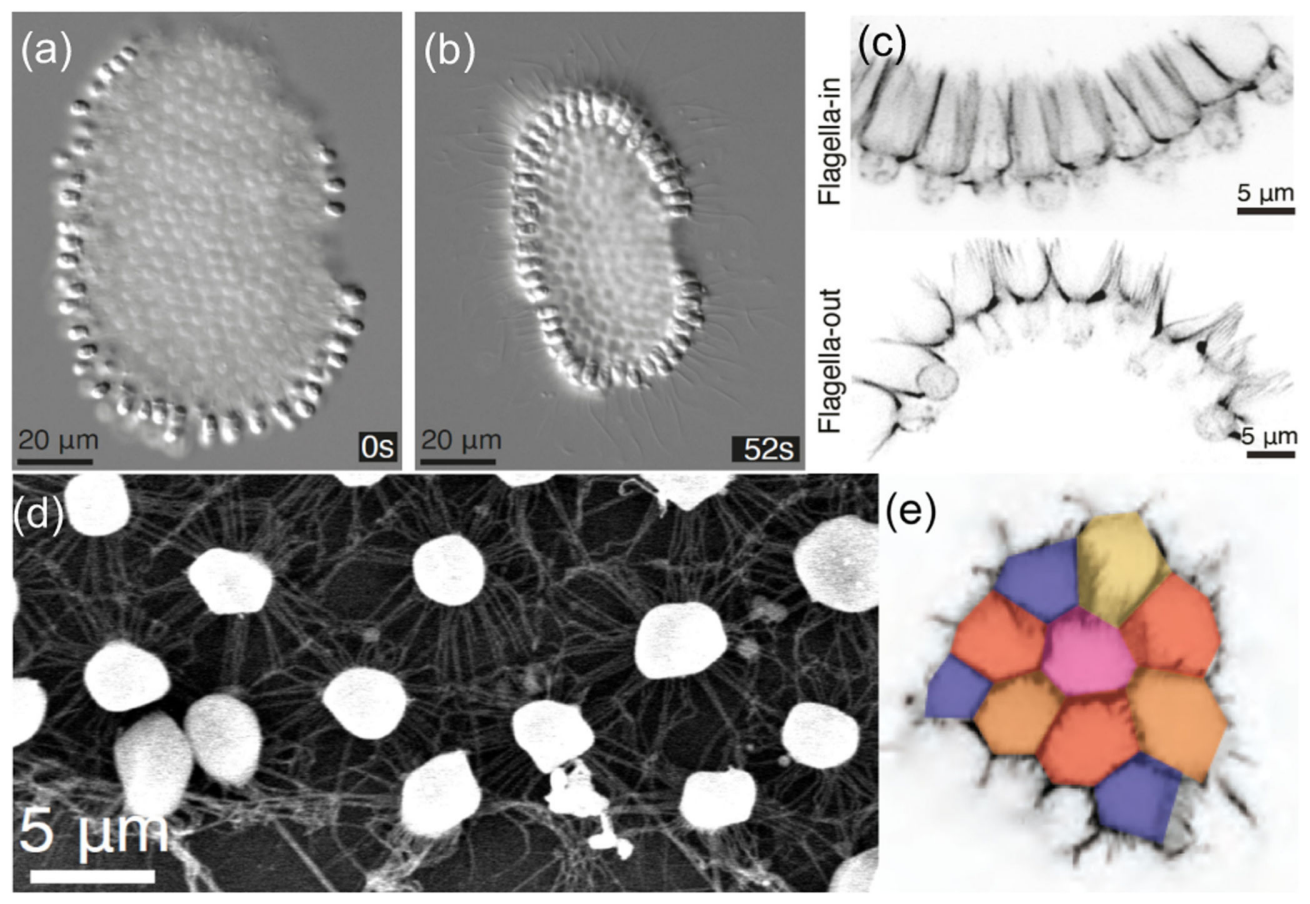
The multicellular choanoflagellate *Choanoeca flexa*. Top views of (a) *flag-in* and (b) *flag-out* states at times relative to removal of light. (c) Close-up of the collar connections in the two states. (d) Electron micrograph showing round white cell bodies connected by microvilli. (e) Confocal slice at the level of microvilli tips, showing their organization into pentagons, hexagons and heptagons. Adapted from [8,10].

**Fig. 2:**
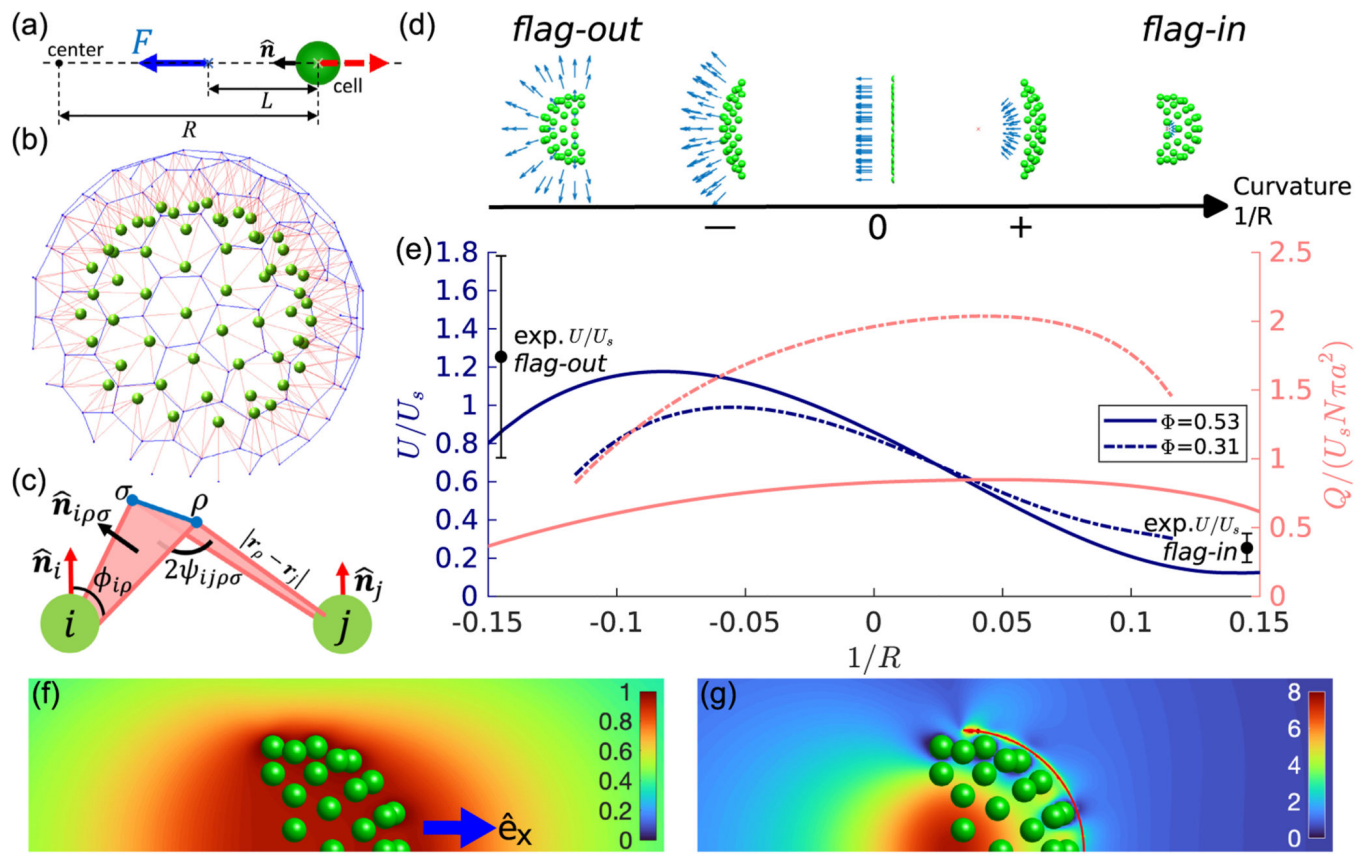
Models for *C. flexa*. (a) Cell body and flagellar force in the *flag-in* state. (b),(c) Mechanical model of interconnecting microvilli in rafts; cells (green spheres, not to scale) are at the vertices of a geodesic icosahedron. Blue arrows indicate flagella forces, red segments represent microvilli, blue dots the microvilli tips, and blue lines the collar-collar interface. (b) Connectivity of the whole raft. (c) Two cells (*i, j*) with apicobasal axis $\left({\hat{n}}_{i},{\hat{n}}_{j}\right)$ connected by filaments meeting at vertices (*ρ, σ*). Effect of curvature 1/*R* on (d) geometry of raft and (e) swimming speed *U* (blue) and flow rate *Q* (red) passing through *S*_*f*_ at constant **Φ**. Solid black circles indicate the experimental *flag-out* and *flag-in* swimming speeds (and uncertainties) [10] relative to the theoretical single cell speed *U*_*s*_. (f),(g) Cross section of the disturbance flows **u**_*d*_ and **u**_*f*_ around the a raft (**Φ** = 0.31, *R* = 8.61) in the reciprocal problems for calculating *U* and *Q*. Heat maps indicate the speed of (f) **u**_*d*_ and (g) **u**_*f*_.

The cells in a *C. flexa* raft are ellipsoidal, with major and minor axes *a* ~ 4 and *b* ~ 3 μm, with a single flagellum of length 2*L* ~ 23 μm and radius *r* ~ 0.5 μm beating with amplitude *d* ~ 2.2 μm and frequency *f* ~ 43 Hz (See experimental results detailed in the Supplemental Material [10]), sending bending waves away from the body. A cell swims with flagellum and collar rearward; the body and flagellum comprise a “pusher” force dipole. From resistive force theory [15] we estimate the flagellar propulsive force to be *F* ~ 2*L*(*ζ*_⊥_ − *ζ*_‖_)(1 − *β*)*fλ* ~ 6.9 pN, where *β* is a function of the wave geometry, *λ* ~ 15 μm is the wavelength [10], *ζ*_⊥_ and *ζ*_‖_ are transverse and longitudinal drag coefficients, *ζ*_⊥_ ~ 2*ζ*_‖_ ~ 4*πμ*/ln(2*L*/*r*), with *μ* the fluid viscosity. These features motivate a computational model in which *N* identical cells in a raft have a spherical body of radius *a* and a point force $F{\hat{n}}_{i}$ acting on the fluid a distance *L* from the sphere center, oriented along the vector ${\hat{n}}_{i}$ that represents the collar axis [Figs. 2(a) and 2(b)]. An idealization of a curved raft involves placing those spheres on a connected subset of the vertices of a *geodesic* icosahedron (one whose vertices lie on a spherical surface) of radius *ρ* ≫ *a*; the area fraction **Φ** of the sheet occupied by cells scales as **Φ** ~ *N*(*a*/*ρ*)^2^. The pentagonal neighborhoods within the geodesic icosahedron serve as topological defects that allow for smooth large-scale surface curvature [16]; confocal imaging shows that a significant fraction (~0.22) of the cellular neighborhoods defined by the microvilli connections are pentagonal, with a smaller fraction of heptagons [Fig. 1(e) and [10]], and earlier work on *C. perplexa* [9] also found nonhexagonal packing. We use the geodesic icosahedron {3, 5+}_(3,0)_ in standard notation [17], with 92 total vertices, and take patches with *N* = 58 to be representative of experimentally observed sizes (10–200 cells). Despite the wide range of sizes, experimental measurements show no correlation between swimming speed and the size of the raft [10]. The vectors ${\hat{n}}_{i}$ point towards (away from) the icosahedron center in the *flag-in* (*flag-out*) forms [Figs. 2(a) and 2(b)]. Deformation of the sheet to a new radius *ρ*′, at fixed **Φ**, requires the new polar angle *θ*′_*i*_ of a cell with respect to the central axis of the sheet be related to its original angle *θ*_*i*_ via *ρ*′^2^(1 − cos *θ*′_*i*_) = *ρ*^2^(1 − cos *θ*_*i*_). We define the scaled force offset length 𝓁 = *L*/*a* ~ 3 and sheet radius *R* = *ρ*′/*a* ≳ 6, and take *R* > 0 in the *flag-in* state.

Images of *C. flexa* [10] show that the packing fraction in the *flag-out* state **Φ**_out_ = 0.47 ± 0.06, less than both the maximum packing fraction $\Phi_{\max}=\pi \sqrt{3}/6≃0.907$. for a hexagonal array of spheres in a plane, and the estimated maximum packing fraction ${\tilde{\Phi }}_{\max}≃0.83$ for circles on a sphere [18]. The packing fraction in the *flag-in* state is **Φ**_in_ = 0.34 ± 0.03, and we use the extremes **Φ**_out_ = 0.53 and **Φ**_in_ = 0.31 to explore the consequences of the differences between the two forms.

Consider an isolated force-free spherical cell at the origin moving at velocity *U*_*s*_**ê**_*x*_ with point force −**F** = −*F***ê**_*x*_ at −*L***ê**_*x*_ acting on the fluid and its reaction force **F** on the cell. The cell experiences Stokes drag −*ζ*_*s*_*U*_*s*_**ê**_*x*_, where *ζ*_*s*_ = 6*πμa*, and drag −*D***ê**_*x*_ arising from the disturbance flow created by the force. By the reciprocal theorem [19], the disturbance drag is *D* = **F** · **ũ**_*d*_ (−*L***ê**_*x*_)/*Û*, where **ũ**_*d*_ (**r**) is the flow created when the cell is dragged along **ê**_*x*_ with unit speed *Û*. Force balance yields the single-cell swimming speed *U*_*s*_ ≡(*F/ζ*_*s*_)[1 − 3/(2𝓁) + 1/(2𝓁^3^)]. Thus, the closer the point force is to the cell (i.e., the smaller is 𝓁), the more drag the cell experiences and the slower is *U*_*s*_. Setting 𝓁 = 3 yields *U*_*s*_ ~ 67 μm/s, comparable to the observed speed of the *flag-out* colony [10] and of fast-swimming cells of the choanoflagellate *S. rosetta* [20].

This intuitive picture extends to a raft of cells. As the raft moves at velocity *U***ê**_*x*_, it experiences a Stokes drag −*ζU***ê**_*x*_. The disturbance flow created by the forces $F{\hat{n}}_{i}$ acting at $r_{i}+L{\hat{n}}_{i}$, produces a drag $D=F\Sigma_{i}{\hat{n}}_{i}\cdot u_{d}\left(r_{i}+L{\hat{n}}_{i}\right)$, where **u**_*d*_ is the (dimensionless) disturbance flow from the raft when it is dragged along **ê**_*x*_ with unit speed. Force balance then yields

(1) $$U=-\frac{F}{\zeta }\sum_{i=1}^{N}{\hat{n}}_{i}\cdot \left[{\hat{e}}_{x}-u_{d}\left(r_{i}+L{\hat{n}}_{i}\right)\right],$$

where $-F\sum_{i}{\hat{n}}_{i}$ is the force propelling the raft along **ê**_*x*_, and **u**_*d*_ has been rendered dimensionless by the unit speed. We compute **u**_*d*_ and *ζ* using a boundary element method [13]. Because of the curved geometry, point forces are closer to neighboring cells in the *flag-in* state than in *flag-out*. Thus, as in Figs. 2(d) and 2(e), for a geometry with a given |*R*|, the *flag-in* state has a larger disturbance drag than the *flag-out* state, and a smaller speed *U*, consistent with experiments (solid black circles).

The difference in swimming speed between the two states can also be explained in terms of $u_{d}\left(r_{i}+L{\hat{n}}_{i}\right)$ in (1). Figure 2(f) shows that **u**_*d*_ inside the raft is close to **ê**_*x*_ because of the curved geometry and screening effects. Hence, *U* is small when the point forces are inside. Meanwhile, **u**_*d*_ outside decays with the distance from the raft, so *U* is large when $r_{i}+L{\hat{n}}_{i}$ is outside. For a given geometry, we expect the swimming speed to be roughly independent of cell number through a balance between the total flagellar force and total drag, a result consistent with our observations in the *flag-out* state [10].

Early work on filter feeding in choanoflagellates focused on the far-field stresslet description [21], but later work showed near-field effects can significantly affect capture rates [22]. To estimate the filter-feeding flux *Q* passing through a colony, without detailed modeling of the microvilli, we measure, in the body frame, the flux passing through the surface *S*_*f*_ projected a distance of 1.2*a* from the cell center along $\hat{n}$, as in Fig. 2(g). By the reciprocal theorem, *Q* can be written in terms of the disturbance flow **u**_*f*_ around a stationary raft and the force **F**_*f*_ on the raft when the surface *S*_*f*_ applies a unit normal pressure $\hat{p}$ on the fluid,

(2) $$Q=F\underset{i}{\sum \;}{\hat{n}}_{i}\cdot u_{f}\left(r_{i}+L{\hat{n}}_{i}\right)+U{\hat{e}}_{x}\cdot \left(F_{f}-\int_{S_{f}}\hat{p}dA\right),$$

where **u**_*f*_ and **F**_*f*_ acquire the units of velocity or pressure and area, respectively, by scaling with $\left|\hat{p}\right|$. Numerical results [Figs. 2(d) and 2(e)] show that the flux due to forces $\sum_{i}F{\hat{n}}_{i}\cdot u_{f}/\hat{p}$ dominates *Q*. Thus, the difference in *Q* between the two states arises from $u_{f}\left(r_{i}+L{\hat{n}}_{i}\right)$ [Fig. 2(g)]. To maintain incompressibility under pressure $\hat{p}$, the disturbance flow **u**_*f*_ is stronger inside the raft than outside. Hence, point forces placed inside the raft pump more flow through the raft than when placed outside.

Figure 2(e) shows the effect of changes in the raft curvature and packing fraction. One value of *R* maximizes swimming speed in the *flag-out* state and another maximizes flux in the *flag-in* state. This arises from a balance between the screening effect mentioned above and alignment of forcing. In the *flag-out* state, an initial decrease in curvature aligns the forces with the swimming direction, increasing swimming speed, but a further reduction in curvature reduces the screening effect as cells are now more spread out in the plane orthogonal to the swimming direction. A similar argument applies to the flow rate maximum in the *flag-in* state. Comparing these maxima, Fig. 2(e) shows that a spread-out colony results in more flux, while a closely packed colony results in faster motility, as seen experimentally [8]. Thus, through the interconversion between the two states, *C. flexa* takes advantage of the hydrodynamic effect of the curved geometry for efficient filter feeding and swimming.

## Mechanics of inversion

Studies suggest that inversion requires an active process within each cell, likely driven by myosin-driven contraction of an F-actin ring at the apical pole [8]. Thus, a full treatment would address the complex problem of elastic filaments responding to the apical actomyosin system and adhering to each other. We simplify this description by considering as in Fig. 2(c) that each cell *i*, located at **r**_*i*_ and surrounded by *m*_*i*_ neighbors, has *m*_*i*_ rigid, straight filaments emanating from it. Two filaments from neighboring cells *i* and *j* meet at vertex *ρ* (or *σ*) located at **r**_*ρ*_ (**r**_*σ*_), with *ϕ*_*iρ*_ the angle between **r**_*ρ*_ − **r**_*i*_ and ${\hat{n}}_{i}$. Two adjacent filaments emanating from cell *i*, and which meet neighboring filaments at vertices *ρ* and *σ*, define a plane whose normal ${\hat{n}}_{i\rho \sigma }$ points toward the apicobasal axis ${\hat{n}}_{i}$. That normal and its counterpart ${\hat{n}}_{j\sigma \rho }$ on cell *j* determine the angle 2*ψ*_*ijρσ*_ between the two planes. The geodesic icosahedron defines the cell positions and the filament network connecting cells. The two sets of angles {*ϕ*} and {*ψ*} are used to define a Hookean elastic energy that mimics the elasticity of the microvilli, allowing for preferred intrinsic angles *ϕ*_0_ and *ψ*_0_ that encode the effects of the apical actomyosin and microvilli adhesion. Allowing for stretching away from a rest length 𝓁_0_, the energy is

(3) $$E=\frac{1}{2}k_{\phi }\underset{i,\rho }{\sum \;}\delta \phi_{i\rho }^{2}+\frac{1}{2}k_{\psi }\underset{i,j,\rho ,\sigma }{\sum \;}\delta \psi_{ij\rho \sigma }^{2}+\frac{1}{2}k_{\ell }\underset{i,\rho }{\sum \;}\delta \ell_{i\rho }^{2},$$

where *δϕ*_*iρ*_ = *ϕ*_*iρ*_ − *ϕ*_0_, *δψ*_*ijρσ*_ = *ψ*_*ijρσ*_ − *ψ*_0_, and 𝓁_*iρ*_ = |**r**_*i*_ − **r**_*ρ*_| − 𝓁_0_. The moduli *k*_*ϕ*_, *k*_*ψ*_ and *k*_*ℓ*_ and quantities *ϕ*_0_, *ψ*_0_ and *ℓ*_0_ are assumed constant for all cells.

The energy (3) is tied to the lattice geometry of the raft. If the cells are in a hexagonal lattice (*m*_*i*_ *=* 6) the system of filaments can achieve *E* = 0 by setting all cell-collar angles to *ϕ*_0_, all collar-collar interface angles to *ψ*_0_, and *ϕ*_0_ = *ψ*_0_. This defines a flat sheet. Increasing *ϕ*_0_ = *ψ*_0_ leads to uniform, isotropic sheet expansion. In a nonplanar raft, curvature is introduced through topological defects (*m*_*i*_ ≠ 6), such as pentagons, and mismatch between the local values of *ψ* and *ϕ*. While pentagonal defects are known to cause out-of-plane buckling in crystal lattices [16], they do not by themselves select a particular *sign* of the induced curvature; there is inherent bistability in the raft that can be biased by changes in the geometry of out-of-plane filaments, akin to the role of “apical constriction” in the shapes of epithelia [23]. This feature of the raft arising as a geometric byproduct of pentagonal defects evokes the concept of “spandrels” in evolutionary biology [24].

For the case of two cells, each with two filaments, and with one vertex between them, if *ϕ* = *ϕ*_0_, *ψ* = *ψ*_0_, and *r* = *𝓁*_0_, then the filament tips lie on a circle of radius *R*_0_ = 1/*C*_0_, where *C*_0_ = sin (*ψ*_0_ − *ϕ*_0_) */𝓁*_0_ sin *ϕ*_0_. While, in general, the equilibrium state of a curved raft will not have *ϕ*_*iρ*_ *= ϕ*_0_, *ψ*_*ijρσ*_ *= ψ*_0_ and *r*_*iρ*_ *=𝓁*_0_ everywhere, we may nevertheless use this relationship to define a proxy for the average raft curvature. Recognizing that in numerical studies stretching effects are small, we ignore variations in *r*_*iρ*_ and define *C* = sin (⟨*ψ*⟩ − ⟨*ϕ*⟩)/𝓁_0_ sin (⟨*ϕ*⟩), where ⟨·⟩ is an average over cells and vertices. The colony is in the *flag-in* (*flag-out*) state when *C* > 0 (*C* < 0).

The simplest model of raft dynamics localizes the viscous drag to the individual cell and vertex positions **r**_*γ*_ and the cell orientation $\hat{n}$ according to gradient flows *ζ*∂_*t*_**r**_*γ*_ = −∂*E/*∂**r**_*γ*_ and $\zeta_{n}\partial_{t}{\hat{n}}_{i}=-(I-\hat{n}\;\hat{n})\cdot \partial E/\partial n_{i}$ driven by the force and torque derived from (3) [25]. Therefore, the dynamical algorithm follows a projected gradient descent [26]. Via a rescaling of time we may set one of the elastic constants to unity (say, *k*_*ϕ*_) and need only consider the ratios *K*_*ψ*_ *= k*_*ψ*_ */k*_*ϕ*_ and *K*_𝓁_ *= k*_𝓁_*/k*_*ϕ*_.

**Fig. 3:**
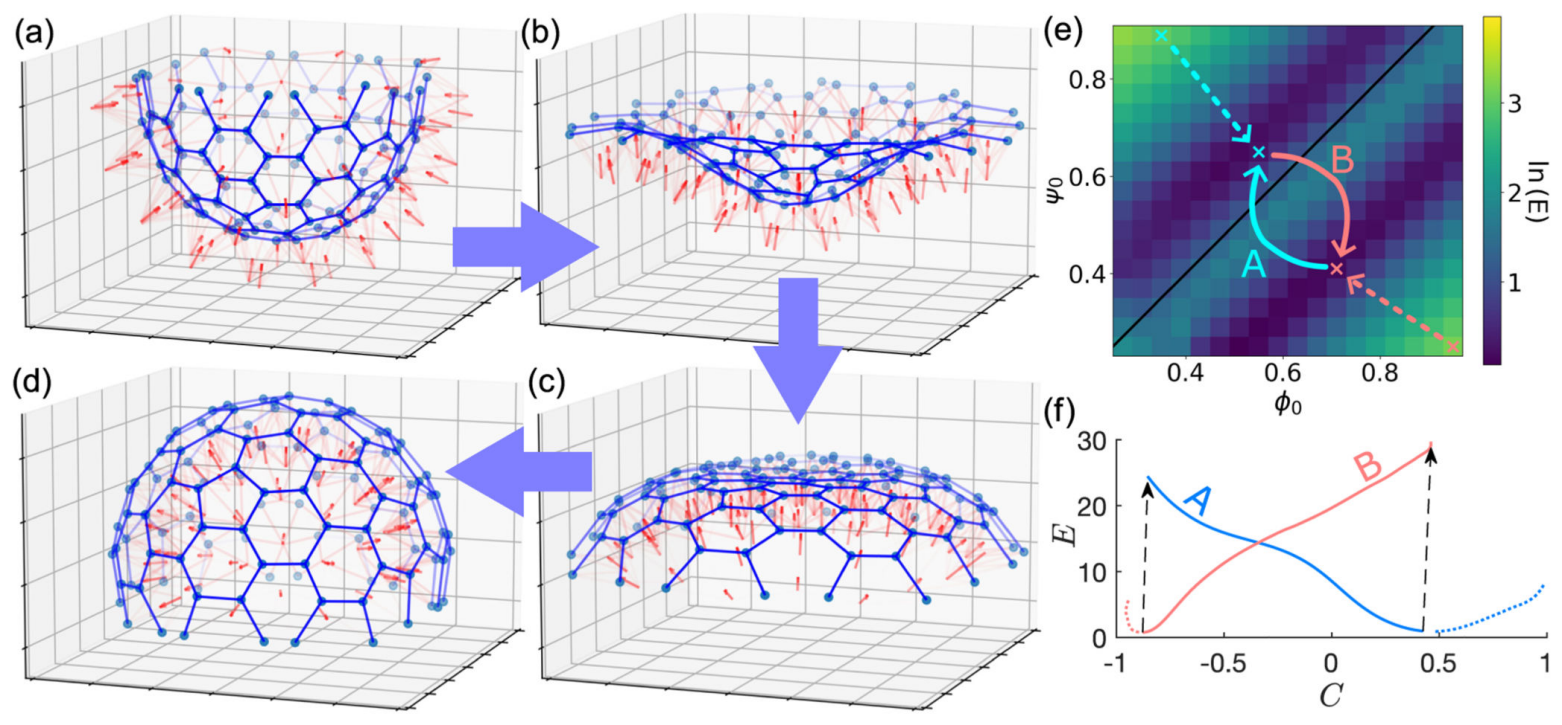
Inversion dynamics from numerical studies. (a)–(d) A colony, initially at a hemispherical minimum with (*ϕ*_0_, *ψ*_0_) = (0.55, 0.65), inverts after a change to (0.71,0.41), with 𝓁_0_ = 0.5, *K*_*ψ*_ = 2 and *K*_*𝓁*_ = 5. Connections between collar vertices are shown in blue, apicobasal axes as red arrows at cell body positions. (e) Residue energy *E* after the colony reaches equilibrium at each preferred angle pair (*ϕ*_0_, *ψ*_0_), where *C* < 0 (*flag-in*) above the black line *ψ*_0_ = *ϕ*_0_, and *C* > 0 (*flag-out*) below. (*f*) Evolution of *E* vs *C* as the colony relax towards a minimum energy state after instantaneous changes in (*ϕ*_0_, *ψ*_0_) shown by the dotted and solid red and blue lines in (*e*).

Figures 3(a)–3(d) show the conversion from *flag-in* to *flag-out*, following an abrupt change in the preferred angles (*ϕ*_0_, *ψ*_0_) that models the fast reaction or relaxation of the F-actin ring in response to a stimulus [10]. This change is path *B* in the space (*ϕ*_0_, *ψ*_0_) in Fig. 3(e), which also shows the line that divides the states and the residue energy *E* after the colony reaches equilibrium at each (*ϕ*_0_, *ψ*_0_). The intermediate shapes exhibit a ring of inflection points similar to those seen in experiments on *C. flexa* and also in the inversion the algae *Pleodorina* [3] and larger species [27,28]. Tracking the energy as each of the two equilibria is achieved, the picture that emerges in Fig. 3(f) is evolution on a double-well potential energy landscape as a biasing field is switched in sign.

We have shown that simple models can explain the swimming, feeding, and inversion of the recently discovered multicellular choanoflagellate *C. flexa* [8]. These results suggest further exploration on a possible continuum description of the sheets, fluid-structure interactions during locomotion, dynamics of photokinesis, and developmental processes of these remarkable organisms.

## Supplementary Material

Fung_etal_SM

Video1

Video2
