# Supplementary material for: Swimming, Feeding, and Inversion of Multicellular Choanoflagellate Sheets
Source: Phys Rev Lett. Author manuscript; Available in PMC 2024 Jun 10. (PMC7616088; doi:10.1103/PhysRevLett.131.168401)
Supplement: Fung_etal_SM [file EMS196598-supplement-Fung_etal_SM.pdf]

Supplemental Material:
Swimming, Feeding and Inversion of Multicellular Choanoflagellate Sheets

Lloyd Fung,[1] Adam Konkol,[1] Takuji Ishikawa,[2] Ben T. Larson,[3]
Thibaut Brunet,[4] and Raymond E. Goldstein[1]

[1] *Department of Applied Mathematics and Theoretical Physics, Centre for Mathematical Sciences, University of Cambridge, Wilberforce Road, Cambridge CB3 0WA, United Kingdom*
[2] *Department of Biomedical Engineering, Tohoku University, 6-6-01 Aoba, Aramaki, Aoba-ku, Sendai 980-8579, Japan*
[3] *Department of Biochemistry & Biophysics, University of California, San Francisco, 600 16th St., San Francisco, CA 94143-2200, USA*
[4] *Department of Cell Biology and Infection, Institut Pasteur, 25-28 rue du Dr. Roux, 75724 Paris Cedex 15, France*

(Dated: September 1, 2023)

This file contains additional experimental results on flagellar dynamics and geometry of *C. flexa*.

I. VIDEO IMAGING AND ANALYSIS

A. Supplementary Videos

C. flexa sheets were imaged in FluoroDishes (World Precision Instruments FD35-100) by differential interference contrast (DIC) microscopy using a 40× (water immersion, C-Apochromat, 1.1 NA) Zeiss objective mounted on a Zeiss Observer Z.1 with a pco.dimax cs1 camera.

Flagellar characteristics reported in Table I were obtained as follows. Beat frequencies were determined by averaging over five cycles for each of twenty randomly selected cells. All other measurements are averages over ten randomly selected cells. The comparatively large wavelength for *flag-out* sheets may be due in part to the fact that the flagellar waveform in that state is not sinusoidal, and its wavelength is thus less well defined than in the *flag-in* state.

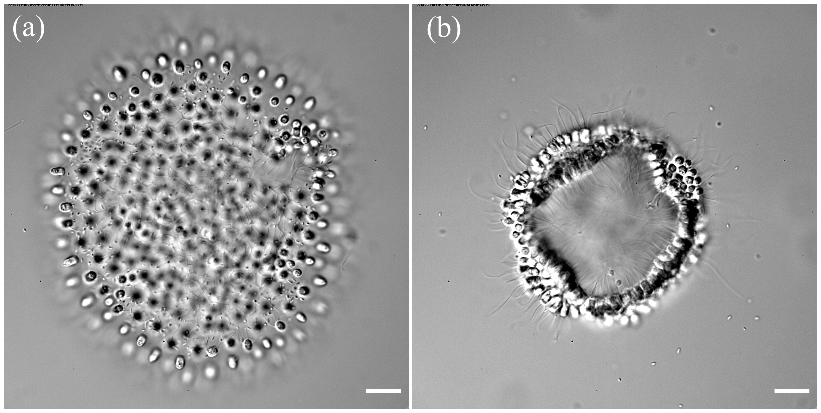

FIG. S1. Flagellar dynamics in *C. flexa* colonies. (a) Snapshot from Video 1, a high speed recording of a *flag-in* sheet used to determine flagellar characteristics reported in Table I. Movie is set to play at 17× slower than real time. (b) As in (a), but for the *flag-out* state in Video 2. Scale bars are 10 μm.

conformation	beat frequency f	length $2L$	amplitude d	wavelength λ	Theoretical single cell swimming speed U_s
flag-in	45 ± 4 Hz	26 ± 5 μm	2.4 ± 0.6 μm	9 ± 3 μm	93 ± 24 μm/s
flag-out	43 ± 8 Hz	23 ± 4 μm	2.2 ± 0.7 μm	15 ± 3 μm	67 ± 20 μm/s

TABLE I. Measurements of flagellar characteristics for the *flag-in* and *flag-out* sheets in Videos S1 and S2, and the theoretical prediction of a single cell swimming speed using Resistive Force Theory, assuming a 4μm radius cell body with a flagella of radius $r = 0.5\,\mu$m and the quantities reported in the table for each state. Uncertainties are standard deviations.

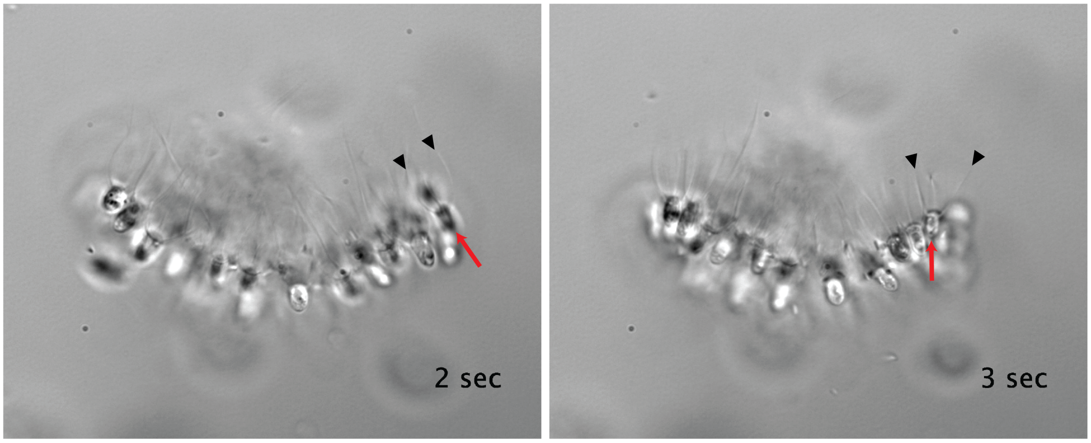

FIG. S2. Collar opening dynamics. Panels show successive images of colony edge as rightmost collar opens. Red arrows indicate cell body, while black triangles indicate tips of collar microvilli.

The speed of opening of microvilli collars was estimated from Supplementary Movie S2 of [S2], which imaged the *flag-in* to *flag-out* inversion process by DIC microscopy. Figure S2 shows that a collar opens in less than 1 s, considerably less than the time for the inversion process itself to go to completion.

B. Estimating the propulsive force from the flagella

In the fluid mechanical model of *C. Flexa* rafts, the action of the flagella on the fluid is modelled as a point force $\mathbf{F}$ acting in the direction $\hat{\mathbf{n}}$. The magnitude F of this force can be approximated using resistive force theory [S1] as

$$F = 2L\left(\zeta_\perp - \zeta_\parallel\right)(1-\beta)f\lambda \tag{S1}$$

where $2L$ is the flagella length, f is the beat frequency, λ is the projected wavelength in the direction of the traveling sinusoidal wave (i.e. $\hat{\mathbf{n}}$), the values of which are listed in Table I, $\zeta_\perp = 4\pi\mu/\ln(2L/r)$ and $\zeta_\parallel = 2\pi\mu/\ln(2L/r)$ are the transverse and longitudinal drag coefficients of a cylindrical filament of radius r, and β is a coefficient that depends on the flagella waveform. In the absence of high-resolution imaging of the waveform, the value of β is approximated by assuming a sinusoidal waveform $f(x)$ with wavelength λ and amplitude d (Table I), as

$$\beta = \frac{\int_0^\lambda \left(1+f'(x)\right)^{-1/2} dx}{\int_0^\lambda \left(1+f'(x)\right)^{1/2} dx}, \tag{S2}$$

which can be found numerically. In the limiting of $2\pi d/\lambda \ll 1$, $\beta \approx 2\pi^2(d/\lambda)^2$.

C. Measuring colony swimming speeds and sizes

Swimming speeds of *C. flexa* sheets were measured using Movie S4 from [S2]. The video starts with sheets under bright illumination, which is then rapidly reduced, triggering colony inversion from *flag-in* to *flag-out*. At the beginning of the movie, nearly all sheets are stuck to the coverslip and immobile, but commence swimming following inversion. Individual sheets were tracked using the TrackMate Fiji plugin [S3, S4] with the following settings: LoG detector threshold 0.1 and radius 50 and Simple LAP tracker with max frame gap 200, linking max distance 150, and gap closing max distance 150. Trajectories were visually inspected, and those from any sheets that remained stuck to the coverslip were excluded from analysis. Speeds were determined from individual trajectories based on frame-to-frame displacements.

To measure colony sizes in terms of projected area, each video frame was segmented using Fiji [S3]. Each frame was first smoothed using the Gaussian blur filter with a 3 pixel radius and then background subtracted using the Subtract Background function. Segmentation was then performed using the Make Binary function.

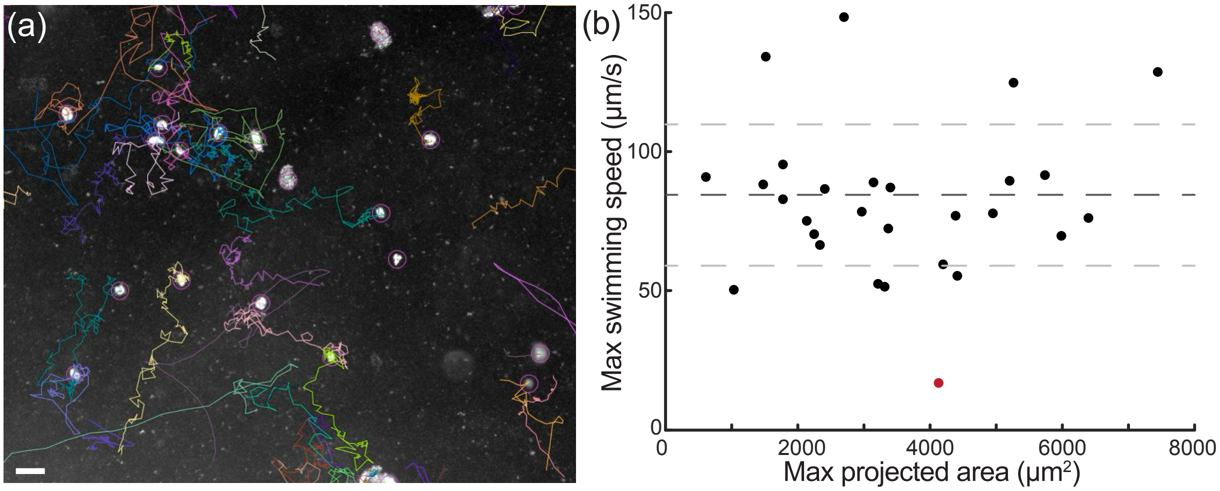

FIG. S3. Swimming speeds of *C. flexa colonies*. (a) Trajectories of swimming colonies tracked using the Trackmate Fiji plugin [S3, S4]. Colored curves represent the trajectories of individual sheets. Scale bar is 50 μm. (b) A plot of colony size in terms of maximum projected area versus maximum frame-to-frame swimming speed. The dark gray dashed line represents the mean maximum swimming speed 84 μm/s, and the light gray dashed lines the standard deviation ± 25 μm/s. The red point represents the single swimming flag-in colony.

II. CONFOCAL IMAGING

Sheets in figures S4 and S5 were fixed and stained with FM1-43FX or with Alexa 488-phalloidin as in [S2]. Sheets were imaged on Zeiss LSM 880 with AiryScan using a 63x, 1.4 NA C Apo oil immersion objective (Zeiss). Z-projections were generated with Fiji [S3]. Packing fraction was estimated by projecting cell bodies located within the same plane in a locally flat portion of the sheet and by manually outlining the border of the colonies (red dotted line in S4).

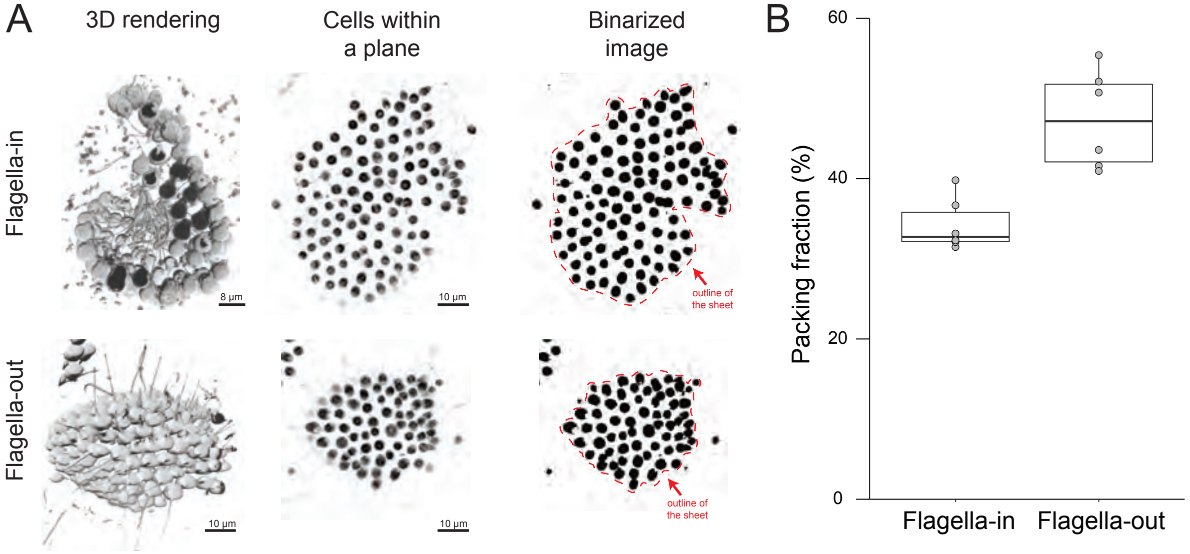

FIG. S4. Packing fraction in flagella-in and flagella-out *C. flexa* colonies. (a) 3D stacks (left column) of sheets stained with the fluorescent membrane marker FM 1-43 FX were imaged by confocal microscopy to determine sheet morphology. Packing fraction was computed by doing a Z-projection of a locally flat portions of individual sheets (middle column) and generating a binarized image in which the area occupied by individual cells appears black (right column). Packing fraction is the ratio of the area occupied by cells to the total colony area within that plane (area within the red dotted line). (b) Boxplot depicting packing fraction values for 6 sheets with flagella out and 6 sheets with flagella-in. p=0.2% by the Mann-Whitney test.

Packing fraction was then computed as the ratio of the area occupied by cells (black area in binarized image in figure

S4) to the total area occupied by the colony (area within the red dotted line in figure S4). Polygonal collar borders in figure S5 were manually outlined and colored with Adobe Illustrator 27.3.1 (2023). Hexagonal and pentagonal outlines were counted in 9 colonies and counts are reported in table II.

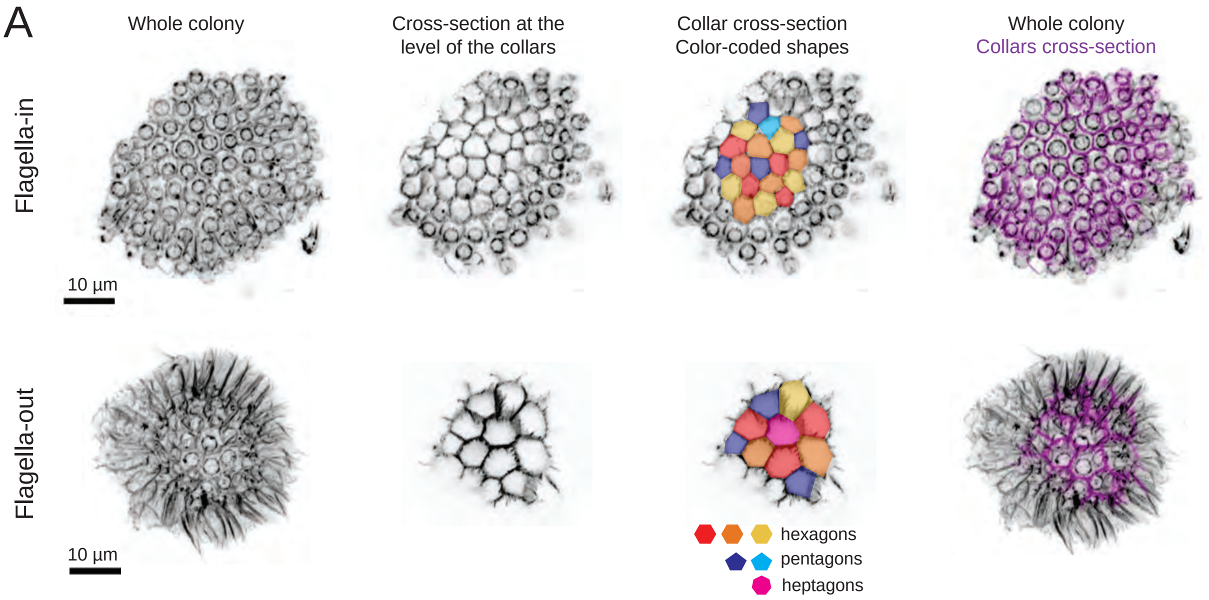

FIG. S5. Hexagonal and polygonal collar outlines within flagella-in and flagella-out *C. flexa* colonies. F-actin within colonies was stained with fluorescent phalloidin, which outlines the microvillous collars linking cells, as well as the actin cytoskeleton within the cell body. Left column: whole-colony Z-projections. Middle left: Z-projections of a few planes intersecting many collars, showing the polygonal outlines of collar contacts. Middle right: color-coded polygons showing a majority of hexagons and a minority of pentagons. Right: whole-colony Z-projection with the plane containing most collars outlined in purple.

TABLE II. Numbers of pentagons, hexagons and heptagons in representative sections of 9 colonies of *C. flexa*.

colony #	1	2	3	4	5	6	7	8	9	total	fraction
pentagons	3	0	1	2	2	4	2	2	3	19	0.22
hexagons	4	3	1	8	7	18	6	7	7	61	0.71
heptagons	1	1	1	0	1	1	0	1	0	6	0.07

[S1] E. Lauga, *The Fluid Dynamics of Cell Motility* (Cambridge University Press, Cambridge, UK, 2020), §7.1.3-7.1.4.

[S2] T. Brunet, B.T. Larson, T.A. Linden, M.J.A. Vermeij, K. McDonald, and N. King, Light-regulated collective contractility in a multicellular choanoflagellate, Science **366**, 326–33 (2019).

[S3] J. Schindelin, I. Arganda-Carreras, E. Frise et al. Fiji: an open-source platform for biological-image analysis. Nat Methods **9**, 676–682 (2012).

[S4] J. Tinevez, N. Perry, J. Schindelin, G.M. Hoopes, G.D. Reynolds, E. Laplantine, S.Y. Bednarek, S.L. Shorte, K.W. Eliceiri, TrackMate: An open and extensible platform for single-particle tracking Methods **115**, 80–90 (2017).
